# S-Nitrosoglutathione Accelerates Recovery from 5-Fluorouracil-Induced Oral Mucositis

**DOI:** 10.1371/journal.pone.0113378

**Published:** 2014-12-05

**Authors:** Maria Adriana Skeff, Gerly A. C. Brito, Marcelo G. de Oliveira, Cintia M. Braga, Matheus M. Cavalcante, Victor Baldim, Rosenilde C. Holanda-Afonso, Carina M. Silva-Boghossian, Ana Paula Colombo, Ronaldo A. Ribeiro, Vivaldo Moura-Neto, Renata F. C. Leitão

**Affiliations:** 1 Laboratory of Cell Morphogenesis, Institute of Biomedical Sciences, Federal University of Rio de Janeiro, Rio de Janeiro, RJ, Brazil; 2 Department of Morphology, School of Medicine, Federal University of Ceará, Fortaleza, CE, Brazil; 3 Institute of Microbiology, Federal University of Rio de Janeiro, Rio de Janeiro, RJ, Brazil; 4 Department of Physiology and Pharmacology, School of Medicine, Federal University of Ceará, Fortaleza, CE, Brazil; 5 Institute of Chemistry, University of Campinas, UNICAMP, Campinas, SP, Brazil; 6 Faculty of Dentistry, University of Grande Rio, Duque de Caxias, RJ, Brazil; 7 Instituto Estadual do Cérebro Paulo Niemeyer, Rio de Janeiro, RJ, Brazil; Seoul St. Mary's Hosptial, Republic of Korea

## Abstract

**Introduction:**

Mucositis induced by anti-neoplastic drugs is an important, dose-limiting and costly side-effect of cancer therapy.

**Aim:**

To evaluate the effect of the topical application of S-nitrosoglutathione (GSNO), a nitric oxide donor, on 5-fluorouracil (5-FU)-induced oral mucositis in hamsters.

**Materials and Methods:**

Oral mucositis was induced in male hamsters by two intraperitoneal administrations of 5-FU on the first and second days of the experiment (60 and 40 mg/kg, respectively) followed by mechanical trauma on the fourth day. Animals received saline, HPMC or HPMC/GSNO (0.1, 0.5 or 2.0 mM) 1 h prior to the 5-FU injection and twice a day for 10 or 14 days. Samples of cheek pouches were harvested for: histopathological analysis, TNF-α and IL-1β levels, immunohistochemical staining for iNOS, TNF-α, IL-1β, Ki67 and TGF-β RII and a TUNEL assay. The presence and levels of 39 bacterial taxa were analyzed using the Checkerboard DNA-DNA hybridization method. The profiles of NO released from the HPMC/GSNO formulations were characterized using chemiluminescence.

**Results:**

The HPMC/GSNO formulations were found to provide sustained release of NO for more than 4 h at concentration-dependent rates of 14 to 80 nmol/mL/h. Treatment with HPMC/GSNO (0.5 mM) significantly reduced mucosal damage, inflammatory alterations and cell death associated with 5-FU-induced oral mucositis on day 14 but not on day 10. HPMC/GSNO administration also reversed the inhibitory effect of 5-FU on cell proliferation on day 14. In addition, we observed that the chemotherapy significantly increased the levels and/or prevalence of several bacterial species.

**Conclusion:**

Topical HPMC/GSNO accelerates mucosal recovery, reduces inflammatory parameters, speeds up re-epithelization and decreases levels of periodontopathic species in mucosal ulcers.

## Introduction

Oral mucositis is considered one of the most common and devastating side effects of chemotherapy and radiotherapy treatment for cancer. Its prevalence ranges from between 10% to 100% depending on system cytotoxicity and patient-associated endogenous variables, therefore representing a significant risk factor for systemic infection [1]–[3].Oral mucositis is an epithelial damage characterized by erythematous, atrophic and ulcerative lesions. Its physiopathology is complex and has been described by Sonis and colleagues as a sequence of interrelated biological events comprising initiation, the primary damage response, signaling and amplification, ulceration, and healing [4].

The more critical phase is the ulcerative phase where bacteria are particularly important. It is well established that both Gram-negative and Gram-positive bacteria form a pseudomembrane that invades the submucosa, which is rich in macrophages, and their cell-wall products (i.e., lipopolysaccharides, lipoteichoic acid, cell wall antigens, and α-glucans) stimulate those cells to further secrete pro-inflammatory cytokines, particularly interleukin-1 (IL-1) and tumor necrosis factor-alpha (TNF-α) [5]. However, thus far, the association between mucositis and changes in the oral bacterial community is poorly understood. The majority of the bacterial species present in the oral cavity are harmless commensal bacteria, and under normal, healthy conditions, they exist in homeostasis in the oral cavity [6]. In patients with malignancies, this homeostasis between host defense and commensal bacterial has been thought to be disturbed by the cancer itself, by cancer-related secondary immunodeficiency, or by prophylactic antibacterial agents. This disruption in homeostasis may contribute to the oral mucosa tissue breakdown following chemotherapy [7]. Cytokines have been shown to stimulate the expression of inducible nitric oxide synthase (iNOS) with consequent production of nitric oxide (NO), a signaling molecule responsible for several physiological and pathophysiological actions throughout the human body, including control of blood flow and modulation of the immune response [8], [9]. Although the chemical structure of NO is simple, its biological effects are indeed complex. This gas appears to play beneficial and detrimental roles. The detrimental effects may include a cytotoxic action towards adjacent host tissues, resulting in pain and tissue lesions. The production of large amounts of NO by iNOS has been show to play a major role in immune reactions and in many inflammatory processes including oral mucositis. Our group has demonstrated that treatment of hamsters with iNOS inhibitors reduced lesions found in 5-fluorouracil (5-FU)-induced oral mucositis significantly, suggesting an important role of iNOS-mediated NO production in the pathogenesis of oral mucositis induced by 5-FU [10]. In contrast, beneficial effects may include antimicrobial activity [11], collagen deposition and keratinocyte proliferation [12]–[14].

Despite its long history and its impact on patients, there are currently no effective treatment options to prevent or treat mucositis associated with chemoradiation therapy for cancer of the head and neck [15]. The goals of mucositis management are to prevent or reduce the severity of toxicity and to manage the associated symptoms, which will, in turn, enable the continued delivery of cancer therapy without interruption or dose reduction and improve the overall prognosis [16].

S-nitrosoglutathione (GSNO) is a primary s-nitrosothiol with strong vasodilatory [17] and wound-healing properties [18], [19] that may release NO spontaneously. It is also capable of transnitrosating cysteine residues of proteins and exerts a marked microbicidal action [20], [21]. These combined properties of GSNO, in addition to its miscibility in non-toxic and mucoadhesive hydroxypropyl methylcellulose (HPMC; a pharmaceutical vehicle) [22], make HPMC/GSNO formulations a promising option for investigating the effects of local NO-donor administration on the course of 5-fluorouracil (5-FU)-induced oral mucositis, which is the purpose of the present study.

## Materials and Methods

### Reagents and drugs

Hydroxypropyl methylcellulose (HPMC; M_n_ 90000), glutathione (GSH), sodium nitrite (NaNO_2_), phosphate-buffered saline (PBS) pH 7.4, acetone and hydrochloric acid (HCl) were purchased from Sigma (St. Louis, MO, USA). 5-fluorouracil (5-FU) was purchased from Roche (Rio de Janeiro, Brazil). All reagents were analytical grade and were used as received. All experiments were performed using deionized water from a Millipore Milli-Q gradient filtration system (Billerica, Massachusetts, USA).

### S-nitrosoglutathione synthesis

S-nitrosoglutathione (GSNO) was synthesized by reacting GSH with NaNO_2_ in equimolar conditions in acidic solution (HCl, pH 2.0) based on a procedure described elsewhere [23]. Solid, dry GSNO was stored protected from light at −20°C for further use.

### Preparation of HPMC/GSNO formulations

Aqueous GSNO solutions (0.10 mM, 0.50 mM and 2.0 mM) were prepared in PBS pH 7.4. Eight milliliters of each solution was added to separate Falcon tubes containing 100 mg of HPMC. The mixtures were vortexed until completely homogenous, thus forming viscous transparent HPMC/GSNO formulations. The falcon tubes were refrigerated for 2 h to assure the complete dissolution of the polymer. The prepared formulations were used for the in vitro and in vivo experiments and were always stored back in the refrigerator after use. The formulations were discarded after 3 days of use. All GSNO solutions and HPMC/GSNO formulations were protected from light during handling using aluminum foil.

### Real-time NO release from the HPMC/GSNO formulations

The profiles of NO release from the HPMC/GSNO formulations (0.1 mM, 0.5 mM and 2.0 mM) were determined over periods of up to 5 h using a chemiluminescence NO analyzer (NOA 280i, Sievers Instruments Inc., Boulder, CO, USA) operated at 37°C with an O2 pressure of 6.0 psig and a N2 pressure of 6.0 Torr. Each replicate of real-time NO release was obtained for a 2-mL volume of the HPMC/GSNO formulation containing 100 µL of an anti-foaming solution (provided by Sievers) in the sampling flask of the instrument. Measurements were performed in triplicate, and the data are expressed as the mean ± SEM.

### *In vivo* studies

#### Animals

Golden hamsters from the Federal University of Ceará weighing 140–200 g were housed in temperature-controlled rooms under 12-hour light-dark cycles, and they received water and food *ad libitum*. Surgical procedures and animal treatments were conducted in accordance with the Guidelines for Institutional and Animal Care and Use of Federal University of Ceará, Brazil. All procedures involving animals were approved by the Federal University of Ceará Committee on the ethical treatment of research animals.

#### Experimental model of 5-FU-induced oral mucositis

Oral mucositis was induced by two intraperitoneal (i.p.) administrations of 5-FU on the first and second days of the experiment (60 and 40 mg/kg, respectively) based on a previously described experimental oral mucositis model [10]. On day 4, under 2.5% tribromoethanol (250 mg/kg, i.p.)-induced anesthesia, the right cheek pouch mucosa was irritated by superficial scratching to potentiate the oral mucositis. The scratching comprised dragging the tip of an 18-gauge needle, twice in a linear manner, across the everted cheek pouch. The animals were euthanized under anesthesia (2.5% tribromoethanol; 250 mg/kg, i.p.) on the 10^th^ or 14^th^ day after the initial injection of 5-FU.

#### Experimental groups

The treated groups were divided into 3 subgroups that differed only in the GSNO concentration of the topical formulation applied to both cheek pouch mucosas twice daily: HPMC formulations containing three different concentrations of GSNO: 0.1 mM (0.1 mM HPMC/GSNO), 0.5 mM (0.5 mM HPMC/GSNO) or 2.0 mM (2.0 mM HPMC/GSNO). All treated animals received 5-FU and mechanical trauma in the right cheek pouch mucosa. The control animals were divided into 4 control subgroups: a group of healthy hamsters that received no treatment (H); hamsters that received only mechanical trauma and topical application of saline swabs to both cheek pouches twice daily (MT); hamsters that received 5-FU, mechanical trauma in the right cheek pouch mucosa and topical application of vehicle to both cheek pouches twice daily (HPMC); and hamsters that received 5-FU, mechanical trauma in the right cheek pouch mucosa and topical application of saline to the mucosa of both cheek pouches (Saline). There were at least 6 animals in each experimental group.

#### Macroscopic analysis of cheek pouches

For macroscopic analysis, inflammatory aspects such as erythema, erosion, vasodilatation, epithelial ulcerations and abscesses, were evaluated in a single-blinded fashion and graded as follows: Score 0, completely healthy cheek pouch with an absence of erosion or vasodilatation; Score 1: presence of erythema but no evidence of erosion in the cheek pouch; Score 2: severe erythema, vasodilation and surface erosion; Score 3: formation of ulcers in one or more faces of the mucosa that did not affect more than 25% of the surface area of the cheek pouch; severe erythema and vasodilatation; Score 4: cumulative formation of ulcers in approximately 50% of the surface area of the cheek pouch; and Score 5: almost complete ulceration of the cheek pouch mucosa. In these cases, fibrosis makes the exposure of oral mucosa difficult [24]. Photographs were used for scoring the lesions.

#### Histopathological analyses

The specimens were fixed in 10% neutral buffered formalin, dehydrated and embedded in paraffin. Sections (5 µm thick) were obtained for hematoxylin-eosin staining (H&E) and were examined by light microscopy (×40). Inflammatory cell infiltration, vasodilatation, presence of hemorrhagic areas, edema, ulcerations and abscesses were determined in a single-blinded fashion and graded as follows: Score 0: normal epithelium and connective tissue without vasodilatation, absent or discreet cellular infiltration, an absence of hemorrhagic areas, ulcerations and abscesses; Score 1: discreet vasodilatation, areas of re-epithelization, discreet inflammatory infiltration with mononuclear prevalence, an absence of hemorrhagic areas, edema, ulcerations and abscesses; Score 2: moderate vasodilatation, areas of hydropic epithelial degeneration, inflammatory infiltration with neutrophil prevalence, the presence of hemorrhagic areas, edema and eventual ulcerations and an absence of abscesses; and Score 3: severe vasodilatation, inflammatory infiltration with neutrophil prevalence, the presence of hemorrhagic areas, edema and extensive ulceration and abscesses [10].

#### Quantification of cytokines by ELISA

Cheek pouch samples were harvested from animals of all groups on the 10^th^ or 14^th^ day after the initial injection of 5-FU for interleukin 1-β (IL-1β; DuoSet ELISA Development kit, R&D systems, #DY501) and tumor necrosis factor α (TNF-α; DuoSet ELISA Development kit, R&D systems, #DY510) measurements. The concentrations of cytokines contained in the samples were measured using an enzyme-linked immunosorbent assay (ELISA), as described previously [25]. The results are expressed as pg/ml of IL-1β or TNF-α.

#### Immunohistochemistry for iNOS, IL-1 β, TNF-α and TGF-β RII

Immunohistochemical staining for iNOS, IL-1β, TNF-α and type II transforming growth factor beta receptor (TGF-β RII) on day 14 of MO was performed using the Envision+Dual Link System HRP method using formalin-fixed, paraffin-embedded tissue sections (4 µm thick) that were mounted on poly-L-lysine-coated microscope slides. The sections were deparaffinized and rehydrated using xylene and graded alcohols. After antigen retrieval (retrieval solution from Spring-ABCAM; 25 min), endogenous peroxidases were blocked (15 min) with hydrogen peroxide (Hydrogen Peroxide Block, Spring-ABCAM) and washed in phosphate-buffered saline (PBS). Non-specific proteins were blocked (20 min) with Protein Block (Spring-ABCAM). Sections were incubated overnight (4°C) with polyclonal goat anti-rabbit primary antibody (anti-iNOS, anti-IL-1β, anti-TNF-α, anti TGF-β RII or anti-Ki67, (Santa Cruz Biotechnology, #sc8310, sc7884, sc1350, sc17791 and sc7846, respectively) diluted 1∶200 in antibody diluent (Dako #S0809). After washing, the slides were then incubated with Dako labeled polymer (Envision Flex Dako #K4010) for 30 min. iNOS, IL-1β, TNF-α or TGF-β RII were visualized using the chromogen 3,3′diaminobenzidine (DAB). Negative-control sections were processed simultaneously as described above but the primary antibody was replaced with antibody diluent (DAKO), and none showed iNOS, IL-1β, TNF-α or TGF-β RII immunoreactivity. Slides were counterstained with Harris hematoxylin, dehydrated in a graded series of ethanol, cleared in xylene and coverslipped. The DAB-stained cells were counted (10 fields per slide; ×1000) for statistical comparisons.

#### Cell proliferation and cell death

5-FU-induced cell death was investigated on the 14^th^ day using the terminal deoxynucleotidyl transferase (TdT)-mediated dUTP nick end labeling (TUNEL) method (ApopTag^R^, #S7101, Merck, Millipore). Briefly, after deparaffinizing, the samples were rehydrated and incubated with 20 µg/mL proteinase K for 15 min at room temperature. Endogenous peroxidases were blocked by treating with 3% (v/v) hydrogen peroxide in PBS for 5 min at room temperature. After washing, the sections were then incubated in a humidified chamber at 37°C for 1 h with TdT buffer containing TdT enzyme and reaction buffer. Specimens were incubated for 10 min at room temperature with a stop/wash buffer and then incubated in the humidified chamber for 30 min with anti-digoxigenin peroxidase conjugate at room temperature. After a series of PBS washes, the slides were covered with peroxidase substrate for color development and then washed in three changes of dH_2_O and counterstained in 0.5% (w/v) methyl green for 10 min. at room temperature. The TUNEL-positive cells were counted (10 fields per slide; ×1000) for statistical comparisons. Cell proliferation was assessed on the 14^th^ day based on Ki67 immunohistochemistry. Ki67 is a nuclear antigen that is present in proliferating cells but absent in quiescent cells [26], [27]. Cheek pouches from control, saline and 0.5 mM HPMC/GSNO groups were immunostained using the streptavidin-biotin-peroxidase method, as described above. The Ki67-positive cells were counted (10 fields per slide; ×1000) for statistical analysis.

#### Microbiological assessment

Just prior to euthanasia, a swab was obtained from the oral ulcers to sample the superficial layer. These samples were submitted to Checkerboard DNA-DNA hybridization with modifications for analysis of the presence and amount of 39 bacterial taxa (**Table 1**) [28], [29]. Immediately after swabbing, the samples were placed in individual Eppendorf tubes containing 150 mL of TE buffer (10 mM Tris-HCl, 0.1 mM EDTA, pH 7.6) and were lysed by adding 150 mL of 0.5 M NaOH. The samples were then boiled for 10 min, and the denatured DNA was neutralized with 800 mL of 5 M ammonium acetate and fixed in individual lanes to a nylon membrane (#RPN303B, Hybond-N1, GE Healthcare Life Sciences, Piscataway, NJ, USA) using the Minislot 30 apparatus (#SB-30, Immunetics, Cambridge, MA, USA). The Miniblotter 45 apparatus (#20930013-1, Immunetics) was used to hybridize whole genomic DNA probes for the bacterial taxa. The probes were labeled with digoxigenin (Random Primer Digoxigenin Labeling Kit, Roche Molecular Systems, Alameda, CA, USA). DNA from serotypes a and b of Aggregatibacter actinomycetemcomitans (Aa) was pooled in one probe. Bound probes were detected using anti-digoxigenin phosphatase-conjugated antibody (#11093274910, Roche Molecular Systems) and fluorescence (#11681982001, ECF, GE Healthcare Life Sciences) by an image capture system (Storm 860, Molecular Dynamics, GE Healthcare Life Sciences). Signals captured on the computer were evaluated visually by comparing with the standards at 105 and 106 cells for each test species. Samples were scored as: 0, not detected; 1, <105 cells; 2, approximately 105 cells; 3, 105–106 cells; 4, approximately 106; and 5,>106 cells. The sensitivity of this assay was adjusted to permit the detection of 104 cells of a given species by adjusting the concentration of each DNA probe. This procedure was performed to provide the same sensitivity for detecting each species. Failure to detect a signal was scored as zero; however, it is possible that counts in the range of 1–1000 were present.

**Table 1:** Bacterial strains used to construct whole genomic DNA probes that were then used for testing hamster buccal epithelial cell samples.

Taxa	Strains	Taxa	Strains
*Aggregatibacter actinomycetemcomitans* a	43718*	*Leptotrichia buccalis*	14201*
*Aggregatibacter actinomycetemcomitans* b	29523*	*Neisseria mucosa*	19696*
*Actinomyces gerencseriae*	23860*	*Parvimonas micra*	33270*
*Actinomyces israelli*	12102*	*Prevotella melaninogenica*	25845*
*Actinomyces odontolyticus*	17929*	*Porphyromonas gingivalis*	33277*
*Actinomyces naeslundii I*	12104*	*Prevotella intermedia*	25611*
*Actinomyces oris*	43146*	*Prevotella nigrescens*	33563*
*Campylobacter rectus*	33238*	*Propionibacterium acnes*	11827*
*Capnocytophaga gingivalis*	33624*	*Selenomonas noxia*	43541*
*Capnocytophaga ochracea*	33596*	*Streptococcus anginosus*	33397*
*Capnocytophaga sputigena*	33612*	*Streptococcus constellatus*	27823*
*Campylobacter showae*	51146*	*Streptococcus mitis*	49456*
*Campylobacter concisus*	484*	*Streptococcus oralis*	35037*
*Eubacterium nodatum*	33099*	*Streptococcus sanguinis*	10556*
*Eikenella corrodens*	23834*	*Streptococcus gordonii*	10558*
*Fusobacterium periodonticum*	33693*	*Streptococcus intermedius*	27335*
*Fusobacterium nucleatum polymorphum*	10953*	*Tannerella forsythia*	43037*
*Fusobacterium nucleatum vincentii*	49256*	*Treponema denticola*	B1†
*Fusobacterium nucleatum nucleatum*	25586*	*Treponema socranskii*	D40DR
*Gemella morbillorum*	27824*	*Veillonella parvula*	10790*

* ATCC (American Type Culture Collection, Rockville, MD);

† The Forsyth Institute, Cambridge, MA.

### *In vitro* studies

#### Cell culture

Oral myofibroblast primary cultures were established from newborn rat (*Rattus norvegicus*) oral mucosas. Briefly, samples of jugal mucous were removed and disinfected in 70% alcoholic solution. The initial fragment was cut into small pieces using an iris scissors and immersed in 0.2% type II collagenase (Invitrogem #17101) and Dulbecco's modified Eagle medium comprising nutrient mixture F12 (DMEM-F12; GIBCO #12400-24) supplemented with glucose (33 mM), glutamine (2 mM), sodium bicarbonate (3 mM), penicillin/streptomycin (0.5 mg/mL), fungizone (2.5 µg/mL) and 10% v/v fetal bovine serum (FBS; Invitrogen #12657-029) at 37°C in a humidified atmosphere containing 5% CO_2_ and 95% air for 1 h. This mixture was centrifuged for 1 min at 1200 rpm, and the pellet containing the cells was resuspended in DMEM-F12. Cells were plated on pre-coated poly-L-lysine plates (5 µg/mL) and the medium was changed every two days until the cells reached confluence.

#### Immunocytochemistry

Cultured oral fibroblasts from newborn rats were incubated in a medium containing GSNO 0.5 mM for 90 min. Cell cultures in a GSNO-free medium were used as a control. Immunofluorescent staining was performed on whole-mount preparations of subconfluent cell cultures using a monoclonal antibody specific for the type of smooth muscle (1∶100; monoclonal mouse anti-human smooth muscle actin clone 1A4I, DAKO #M0851), anti-phalloidin conjugated to fluorescein (FITC 1∶200; SIGMA #P5282) and mouse anti-human Ki67 (1∶50; monoclonal mouse anti-human, NOVOCASTRA #NCL-ki67-MM1). For immunocytochemical analysis, 2×105 cells were plated on coverslips placed in 24-well plates. The cells were fixed with 4% PFA in PBS for 15 min. Fixed cells were then washed with PBS and incubated with 5% BSA diluted in PBS for 30 min. Cells were incubated with mouse anti-human Ki67 (1∶100), anti-phalloidin and anti-smooth muscle alpha actin antibodies. Cells were incubated overnight at 4°C with the primary antibodies, then washed with PBS and incubated with secondary antibodies conjugated to monoclonal Alexa Fluor 488 (donkey anti-goat;1∶500; FITC; INVITROGEN) or 546 (goat anti-mouse; 1∶500; rhodamine; INVITROGEN) for 2 h. Cells were then washed with PBS, stained with DAPI (SIGMA #D9542), washed with PBS and mounted. Negative controls were performed using non-immune mouse IgG. Cells were imaged using an epifluorescence microscope (Nikon TE300) and a confocal microscope (Leica TCS-SP5) equipped with a 63× NA 1.40 oil-immersion objective. Image processing was performed using CorelDRAW Graphics Suite X5. The Ki67-positive cells were counted (10 fields/slide; 1000×) for statistical comparisons.

### Statistical analysis

The data were presented as mean ± SEM or as median where appropriate. Kruskal Wallis and Mann Whitney tests were used to compare means and medians using Prism 6 (GraphPad Software Inc., La Jolla, CA, USA). The microbial analyses were carried out using the SPSS program v. 19.0 (IBM, Armonk, New York, NY, USA). The microbiological data were presented as mean levels (×10^5^ bacterial cells) of the tested species. The levels of each species were calculated by transforming the scores 0–5 in counts. Then, mean counts were computed for each animal (5 per group) and within groups. The mean frequency (prevalence) of detection of a studied species was also obtained for each group. Significant differences among groups, for levels and prevalence, were determined by Kruskal-Wallis and Mann Whitney tests, for comparisons including all groups or between two groups, respectively. In the in vitro study, each experiment was performed in three replicates and the results are the means ± SD of three independent experiments. All the values were represented as the means ± standard deviation and statistical analysis was performed with the use of the Mann-Whitney test (control group versus 0.5 mM GSNO treated group). The significance level was 5% for all analyses.

## Results

### Profiles of NO release from the HPMC/GSNO formulations

**Figure 1:**
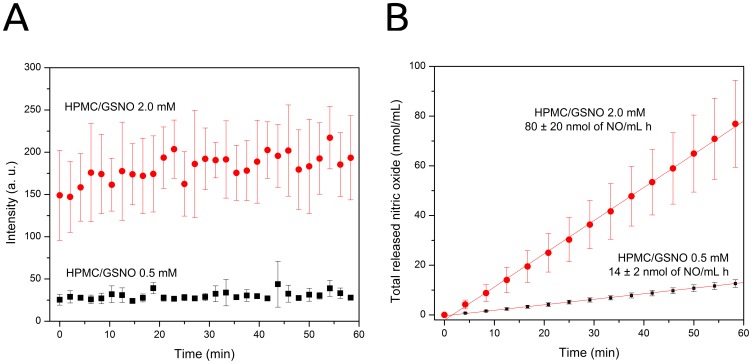
Real-time NO release profiles of the HPMC/GSNO formulations. (A) Kinetic curves of NO release from the 0.5 mM and 2.0 mM HPMC/GSNO formulations, measured by chemiluminescence. (B) Integrated NO signals extracted from the curves of Fig. 1A, which indicate the total NO released from the formulations over the same time-scale. The straight lines denote linear regressions of the experimental data.

Figure 1A shows that the 0.5 and 2.0 mM HPMC/GSNO formulations released NO in a concentration-dependent and sustained fashion for 1 h. The level of NO released from the 0.1 mM HPMC/GSNO formulation was below the detection limit of the chemiluminescence method.

Figure 1B shows the corresponding integrated NO signals extracted from the two curves in Figure 1A which represent the total amount of NO released from these formulations on the same time scale. The rates of NO release shown above the curves in Fig. 1B were obtained by linear regression of the experimental data (denoted by the straight lines). The total amounts of NO released from the 2.0 mM and 0.5 mM HPMC/GSNO formulations after 1 h were 4% and 3% of initial GSNO content, respectively.

Complementary measurements of NO release from the 2.0 mM HPMC/GSNO formulation in a prolonged time showed that it can maintain a constant rate of NO release for 4 h at 37°C (**Figure S1 in** **File S1**). This rate corresponds to a total amount of 320±80 nmol of NO released per mL of hydrogel, implying that after 4 h ca. 12% of the initial pool of GSNO in the gel was decomposed. An extrapolation to longer times allows considering that the biological action of NO in topical applications is probably maintained over 12 h, although a gradual decrease in the rate of NO release is expected in this period. Of course, in oral applications of a hydrogel other important factors must be considered over long periods, especially the dissolution of the hydrogel by the saliva and its mechanical removal by the fluid dynamics of the oral cavity. In any case, the desired biological action can be modulated by establishing the ideal frequency of reapplication of the hydrogel.

### Macroscopic and histopathological analyses of oral mucosa

**Figure 2:**
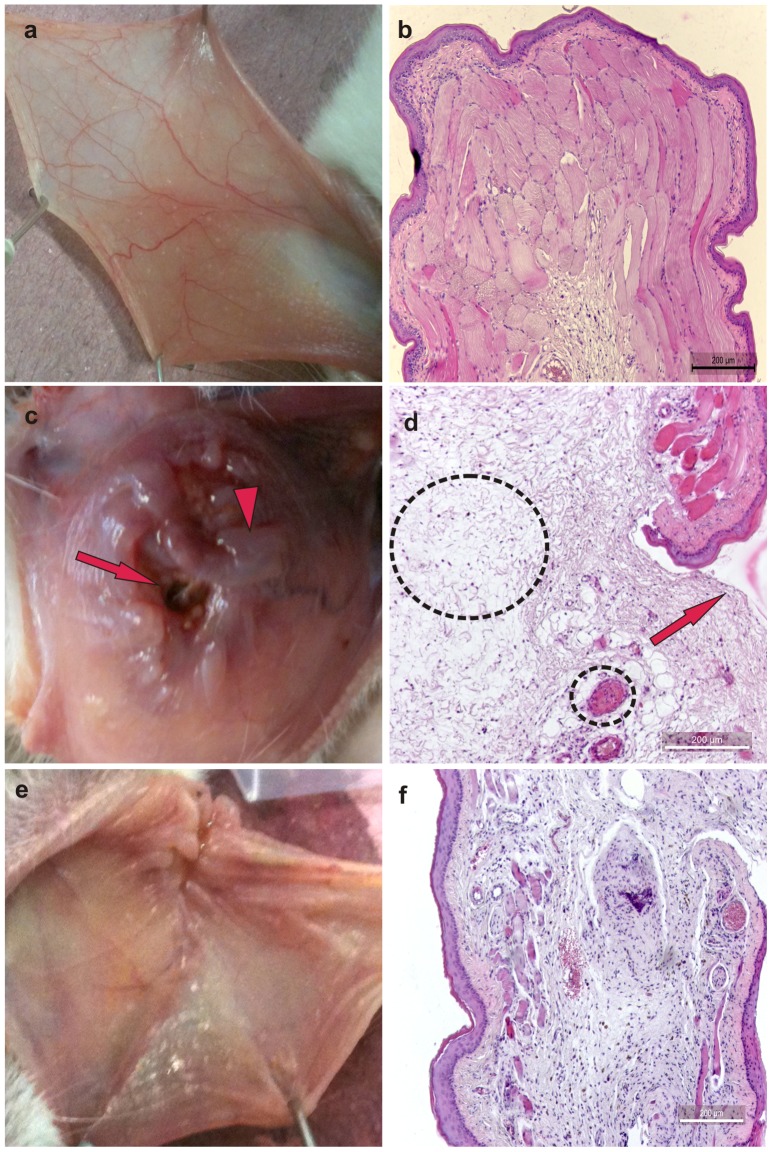
Macroscopic and microscopic aspects of healthy hamster cheek pouches (a and b) or cheek pouches from animals subjected to 5-fluorouracil (5-FU)-induced oral mucositis and that received topical applications of the vehicle HPMC (c and d) or GSNO (0.5 mM) (e and f) observed on day 14. Oral mucositis was induced by i.p. dministration of 5-fluorouracil followed by mechanical trauma of the cheek pouch. Animals received topical applications of S-nitrosoglutathione (0.5 mM HPMC/GSNO) or vehicle only (HPMC) at 1 h prior to 5-FU and every 12 h thereafter for 14 days. Each cheek pouch was everted and photographed, and samples were removed and processed for hematoxylin and eosin staining (100× magnification). Details: in Figure 2c, macroscopic edema (arrowhead) and vasodilation (dotted line) are shown; in Figure 2d, microscopic edema (larger circled) and severe vasodilation (smaller circled) are shown. Arrow = ulcerations.

The i.p. administration of 5-FU followed by mechanical trauma of the cheek pouch caused significant macroscopic lesions (P<0.05), which were observed on day 10. Compared with the control group of healthy animals (**Figure 2a** **;** **Table 2**) or animals subjected to mechanical trauma, only 5-FU and mechanical trauma (**Table 2**) caused increased erythema, hemorrhage, extensive ulcers and abscesses (**Table 2**). These effects of 5-FU at day 10 were not inhibited significantly by topical application of HPMC/GSNO at any of the concentrations tested (**Table 2**). On day 14, treatment with 0.5 mM HPMC/GSNO (**Figure 2e** **;** **Table 2**) significantly (P<0.05) reduced inflammatory alterations when compared with the non-treated group subjected to the experimental mucositis (5-FU administration and mechanical irritation) and that received topical application of HPMC only (**Figures 2c** **;** **Table 2**).

**Table 2:** Macroscopic and microscopic analysis of cheek pouch tissue of hamsters subjected to 5-FU-induced oral mucositis and treated with topical applications of S-nitrosoglutathione (0.5 mM), observed on days 10 and 14.

Control and experimental groups	H	MT (10^th^ day)	MT (14^th^ day)	5-FU					
				HPMC (10^th^ day)	HPMC (14^th^ day)	Saline (10^th^ day)	Saline (14^th^ day)	0.5 mM (10^th^ day)	0.5 mM (14^th^ day)
**Macroscopic analysis**	0(0–0)	3(0–4)*	2(2–4)*	3(2–5)*	3(2–5)*	4(2–5)*	3(2–5)*	4,5(1–5)*	2(1–5)*,*
**Microscopic analysis**	0(0–0)	2.5(0–3)*	1.5(0–3)*	3(1–3)*	2(1–3)*	2.5(0–3)*	3(2–3)*	3(0–3)*	1(0–3)*,**

Oral mucositis was induced in hamsters by intraperitoneal (i.p.) injection of 5-FU followed by mechanical trauma (MT) of the cheek pouch. Animals received topical applications of S-nitrosoglutathione (0.5 mM), vehicle (HPMC) or saline 1 h prior to 5-FU and every 12 h thereafter for 10 days. H = healthy group, i.e., animals without oral mucositis. Data denote the median values (and range) of macroscopic or microscopic scores in six animals per group.

*P<0.05 compared with healthy animals (H group),

**P<0.05 compared with Saline group. Data were analyzed using Kruskal Wallis and Mann Whitney tests.

Histopathology of the cheek pouches of animals subjected to 5-FU-induced oral mucositis revealed accentuated vasodilatation, intense cellular infiltration with neutrophil prevalence, hemorrhagic areas, edema, abscesses and extensive ulcers at day 10, compared with the cheek pouches of healthy hamsters (**Table 2**) or hamsters exposed to mechanical trauma only (**Table 2**). The topical application of HPMC/GSNO did not significantly inhibit 5-FU-induced inflammatory cell infiltration, edema, abscesses, hemorrhage or ulceration at day 10 of the experiment (**Table 2**). Figure S2 in file S1 illustrates the neutrophil infiltration (by the quantification of the neutrophils in the cheeh pouch) in the seven experimental groups on the 14^th^ day. The treatment with 0.5 mM HPMC/GSNO significantly reduced the number of neutrophils in the cheek pouches as compared to HPMC group (**Figure S2 in** **File S1**). In contrast, we observed an increased re-epithelization of the cheek pouch of animals treated with topical applications of 0.5 mM HPMC/GSNO on the 14^th^ day of the experiment (**Figure 2f** **;** **Table 2**) when compared with the non-treated group (**Figure 2d** **;** **Table 2**), and no effect of the other concentrations was observed.

### Cheek pouch tissue cytokine levels

**Figure 3:**
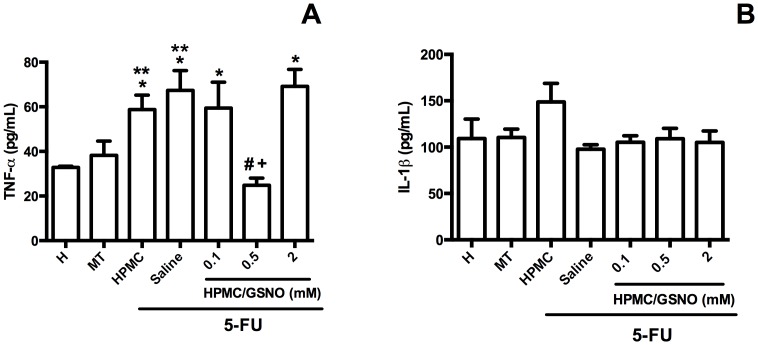
TNF-α and IL-1β levels (pg/ml) in the cheek pouches of hamsters subjected to 5-FU-induced oral mucositis on day 14. Oral mucositis was induced in hamsters by intraperitoneal (i.p.) injection of 5-FU followed by mechanical trauma (MT) of the cheek pouch. Animals received topical applications of a gel containing S-nitrosoglutathione (0.1, 0.5 and 2.0 mM HPMC/GSNO) 30 min prior to 5-FU and twice daily thereafter for 10 days or 14 days. Control groups comprised normal animals (N), animals subjected to mechanical trauma (MT) only and animals subjected to 5-FU-induced oral mucositis that received local application of saline (saline) or vehicle (HPMC). Bars denote the mean ± standard error of TNF-α and IL-1β levels in six animals per group. *denotes values significantly different (P<0.05) from the Healthy group; +denotes values significantly different (P<0.05) from the Saline group; #denotes values significantly different (P<0.05) from the HPMC group. Data were analyzed using the Kruskal Wallis and Mann Whitney tests.

5-FU significantly (P<0.05) increased cheek pouch tissue levels of TNF-α but not IL-1β on day 14 (**Figures 3A and 3B**). The topical application 0.5 mM HPMC/GSNO but not 0.1 mM or 2.0 mM HPMC/GSNO reduced the 5-FU-induced increase in TNF-α and restored this parameter to the levels of Healthy or MT control animals (**Figure 3A**). No significant differences in IL-1 levels were observed between experimental groups on day 14 (**Figure 3B**).

### Immunohistochemical staining for iNOS, IL1-β, TNF-α, TGF-β RII and Ki67

**Figure 4:**
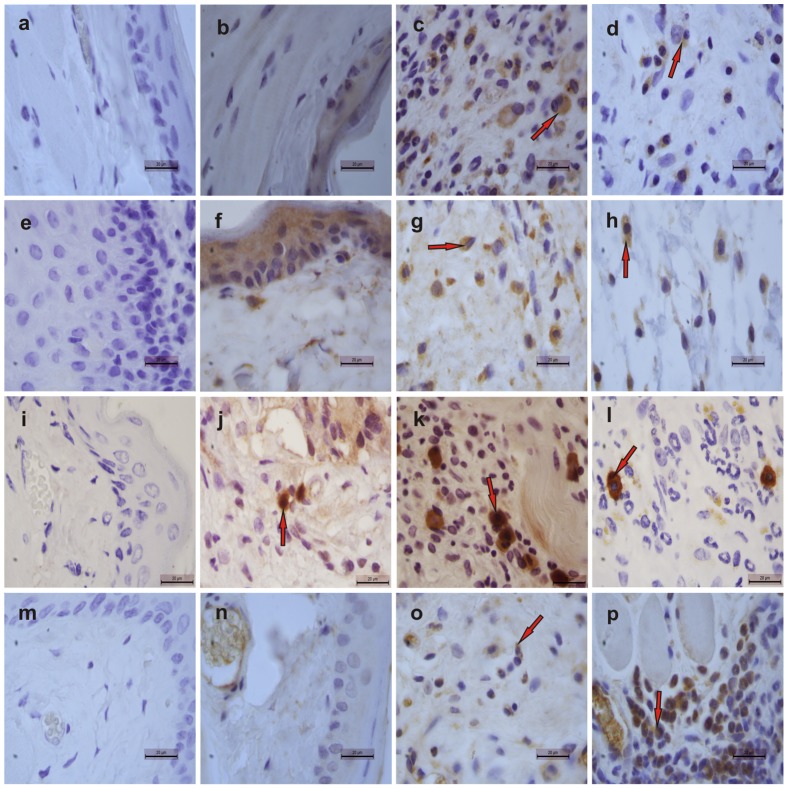
Representative examples of iNOS (1^st^ row), IL-1β (2^nd^ row), TNF-α (3^rd^ row) and TGF-β RII (4^th^ row) immunostaining on day 14 in tissues from cheek pouches of hamsters subjected to 5-FU-induced oral mucositis. Staining was performed using cheek pouches from healthy animals (b, f, j, n) and animals subjected to 5-FU-induced mucositis that received topical applications of S-nitrosoglutathione (GSNO; 0.5 mM; d, h, l, p) or saline (c, g, k, o). Negative controls were samples of cheek pouches where the primary antibody was replaced with PBS-BSA (5%); no immunostaining was detected (a, e, i, m). Magnification, ×1000.

**Figure 5:**
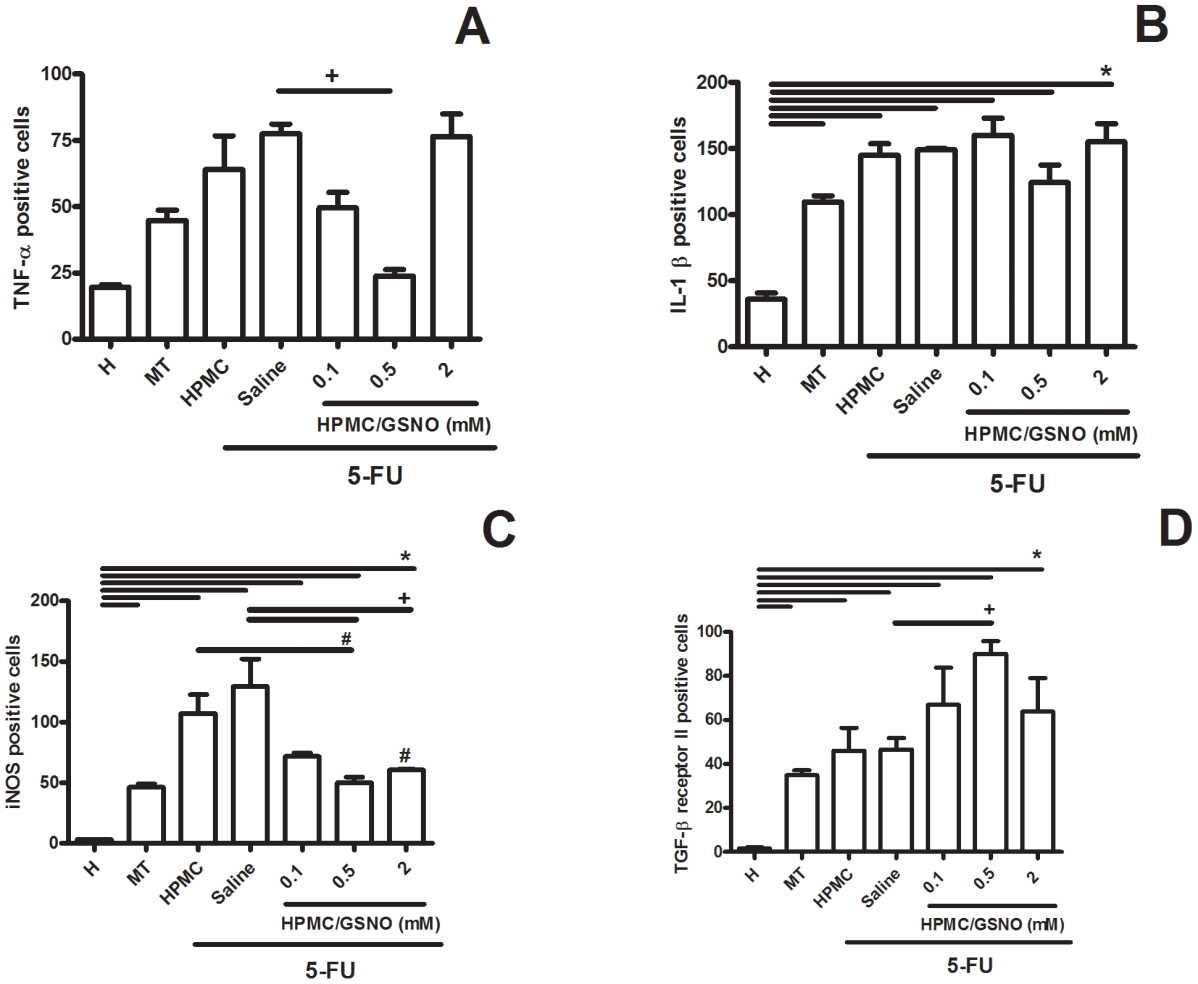
Quantification of TNF-α- (A), IL-1β- (B), iNOS- (C) and TGF-β RII - (D) positive cells in cheek pouch tissues of hamsters subjected to 5-FU-induced oral mucositis, on day 14. Oral mucositis was induced in hamsters by intraperitoneal (i.p.) injection of 5-FU followed by mechanical trauma (MT) of the cheek pouch. Animals received topical applications of a gel containing 0.5 mM S-nitrosoglutathione (GSNO) 30 min prior to 5-FU and twice daily thereafter for 10 days or 14 days. Control groups comprised healthy animals (H) and animals subjected to 5-FU-induced oral mucositis that received local applications of saline (Saline). Cells positive for staining were counted (10 fields per slide, 400×) for statistical comparisons. Bars denote the means ± standard errors of positive cells from four slides per group (4 animals per group). *denotes significant differences (P<0.05) compared with the Healthy group; +denotes a significant difference (P<0.05) compared with the Saline group; #denotes a significant difference (P<0.05) compared with the HPMC group. Data were analyzed using the Kruskal Wallis and Mann Whitney tests.

**Figure 6:**
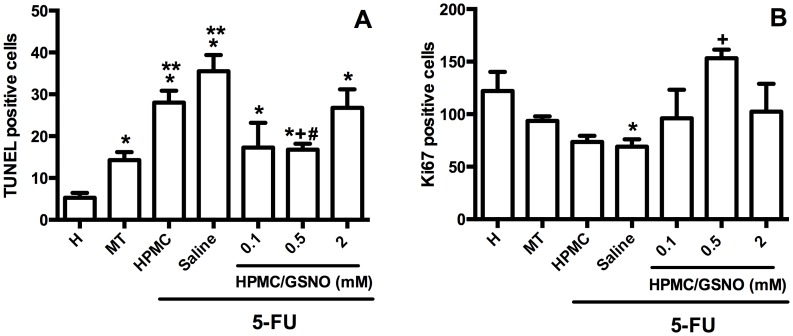
Cell death and proliferation in the cheek pouches of hamsters subjected to 5-FU-induced oral mucositis, on day 14. Oral mucositis was induced in hamsters by intraperitoneal (i.p.) injection of 5-FU followed by mechanical trauma (MT) of the cheek pouch. Animals received topical applications of a gel containing 0.5 mM S-nitrosoglutathione (GSNO) 30 min prior to 5-FU and twice daily thereafter for 10 days or 14 days. Control groups comprised healthy animals (H) and animals subjected to 5-FU-induced oral mucositis that received local applications of saline (saline). The TUNEL- and Ki67-positive cells were counted (10 fields per slide, 400×) for statistical comparisons. Bars denote the means ± standard errors of stained cells from at four slides per group (4 animals per group). *denotes a significant difference (P<0.05) compared with the Healthy group; **denotes a significant difference (P<0.05) compared with the MT group, +denotes a significant difference (P<0.05) compared with the Saline group; #denotes a significant difference (P<0.05) compared with the HPMC group. Data were analyzed using the Kruskal Wallis and Mann Whitney tests.

Figure 4 illustrates immunostaining for iNOS, IL-1β, TNF-α and TGF-β RII in cheek pouches of hamsters subjected to 5-FU-induced oral mucositis (**Figures 3c, 3g, 3k and 3o**, **respectively**) on the 14th day of treatment compared with the weak staining observed in the Healthy control group (**Figures. 3b, 3f, 3j, 3n**). Local application of 0.5 mM HPMC/GSNO for 14 days caused a considerable reduction in both iNOS and TNF-α immunostaining (**Figures 3d and 3i**, **respectively**) but had no effect on IL-1β immunostaining (**Figure 4h**) when compared with the untreated group comprising animals subjected to experimental mucositis and that received saline (**Figures 3c and 3k** **, respectively**). In contrast, 0.5 mM HPMC/GSNO increased TGF-β RII immunostaining in cheek pouch tissue markedly (**Figure 4p**) compared with the Saline group (**Figure 4o**). When the antibodies were replaced with PBS/BSA 5%, no immunostaining was detected (**negative control;** **Figures 3a, 3e, 3i and 3m**). Figure 5 shows the quantification of cells in cheek pouches of hamsters subjected to 5-FU-induced oral mucositis that received daily applications of saline (Saline) or 0.5 mM HPMC/GSNO for 14 days that were positive for TNF-α (**Figure 5A**), IL-1β (**Figure 5B**), iNOS (**Figure 6C**) or TGF-β RII (**Figure 5D**). A significantly greater number of TNF-α-, IL-1β- and iNOS-positive cells (P<0.05) were observed in cheek pouches of animals subjected to oral mucositis and that received saline or HPMC when compared with the Healthy group (**Figures 4A, 4B and 4C** **, respectively**). The topical application of 0.5 mM HPMC/GSNO reduced (P<0.05) the number of iNOS- and TNF-α-positive cells when compared with both the HPMC and Saline groups; however, the treatment did not reduce the number of IL-1β-positive cells (**Figure 5B**). Experimental oral mucositis significantly (P<0.05) increased TGF-β RII -positive cells in cheek pouch tissue compared with the Healthy group. There was also a 2-fold increase (P<0.05) in the number of TGF-β receptor-II-positive cells in the 0.5 mM HPMC/GSNO group compared with the HPMC and Saline groups.

### Cell proliferation and cell death

The cheek pouches of animals subjected to 5-FU-induced oral mucositis showed a significant increase (P<0.05) in TUNEL-positive cells on day 14 when compared with the Healthy control group. 0.5 mM HPMC/GSNO treatment substantially (P<0.05) reduced the number of TUNEL-positive cells in cheek pouch tissue compared with both the HPMC and Saline groups (**Figure 6A**). Figure 5B illustrates cell proliferation (based on Ki67 expression) in cheek pouches of hamsters subjected to oral mucositis. On day 14, a significant decrease in the number of Ki67-positive cells in the Saline group compared with the Healthy group was observed. Treatment of animals subjected to 5-FU-induced mucositis with 0.5 mM HPMC/GSNO for 14 days resulted in a 2-fold increase in Ki67-positive cells compared with the HPMC and Saline groups.

### Microbiological assessment

**Figure 7:**
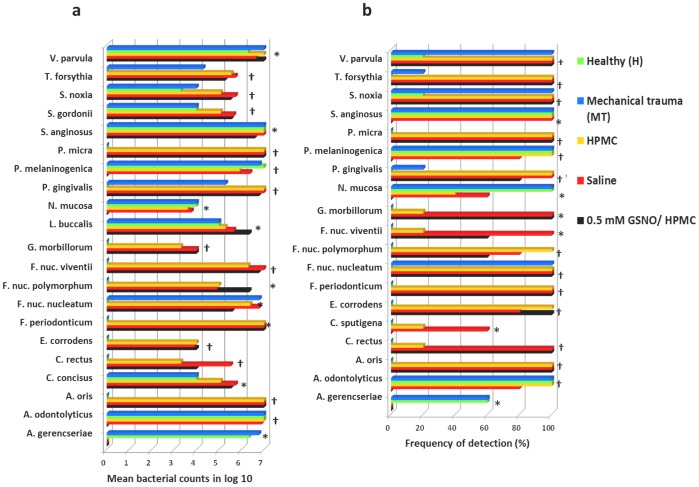
Oral bacterial species evaluated in mucositis lesions of hamsters. Only species showing significant differences between groups are displayed. Left panel: mean bacterial counts using log 10; Right panel: frequency of detection, %. * denotes p<0.05 and † p≤0.001 between groups using the Kruskal Wallis test. The number of animals in each group was at least five.

The prevalence and the mean bacterial levels in the swabs obtained from the oral ulcers of the animals are presented in Fig. 7. Of all 39 bacterial genomic probes tested, only 21 and 19 species showed significant differences between groups in levels and frequency of detection, respectively (p<0.05, Kruskal-Wallis test). *Actinomyces naeslundii* I, *Streptococcus constellatus*, *Eubacterium nodatum*, *Aggregatibacter actinomycetemcomitans*, *Treponema socranskii*, *Streptococcus sanguinis*, *Streptococcus oralis*, *Capnocytophaga ochracea*, *Actinomyces israelii*, *Treponema denticola*, *Prevotella nigrescens*, *Campylobacter showae*, and *Capnocytophaga gingivalis* were not detected in any sample suggesting that they do not inhabit the oral cavity of hamsters under any circumstances. Conversely, *Streptococcus intermedius*, *Streptococcus gordonii*, *Propionibacterium acnes*, *Leptotrichia buccalis*, *Campylobacter concisus*, and *Prevotella intermedia* were detected in all samples. Comparisons between Healthy and MT groups showed similar prevalences of all species analyzed with the exception of *Fusobacterium nucleatum nucleatum*, *Selenomonas noxia*, and *Veilonella parvula*, which showed a lower prevalence in the Healthy group (p<0.05). These data suggest that mechanical trauma of cheek pouch tissue itself may affect the composition of oral microbiota because the MT group did not undergo 5-FU administration. However, chemotherapy led to a significant change in the composition of the microbial community and resulted in a significant increase in the levels and/or prevalence of several species (*Porphyromonas gingivalis*, *Fusobacterium nucleatum vicentii*, *Campylobacter rectus*, *Parvimonas micra*, *Actinomyces oris*, *Fusobacterium nucleatum polymorphum*, *Fusobacterium periodonticum*, *S. gordonii*, *Tannerella forsythia*, *S. noxia*, *Eikenella corrodens*, *Gemella morbillorum*, *L buccalis*, and *C. concisus*) compared with the MT group and the Healthy group (p<0.05). In contrast, *Actinomyces gerencseriae*, *Neisseria mucosa*, and *Prevotella melaninogenica* were present at significantly lower levels in the groups subjected to 5-FU treatment (0.5 mM HPMC/GSNO, Saline, and HPMC groups) compared with the MT and H groups (p<0.05).

Comparisons between the 0.5 mM HPMC/GSNO and Saline or HPMC groups revealed that the prevalence and levels of only two or four species, respectively, showed significant differences. Levels of *P. melaninogenica* and *Actinomyces odontolyticus* were significantly higher in the Saline and HPMC groups compared with the 0.5 mM HPMC/GSNO group (p<0.05) suggesting an antibacterial effect of GSNO.

### Immunocytochemistry of oral fibroblast primary culture

**Figure 8:**
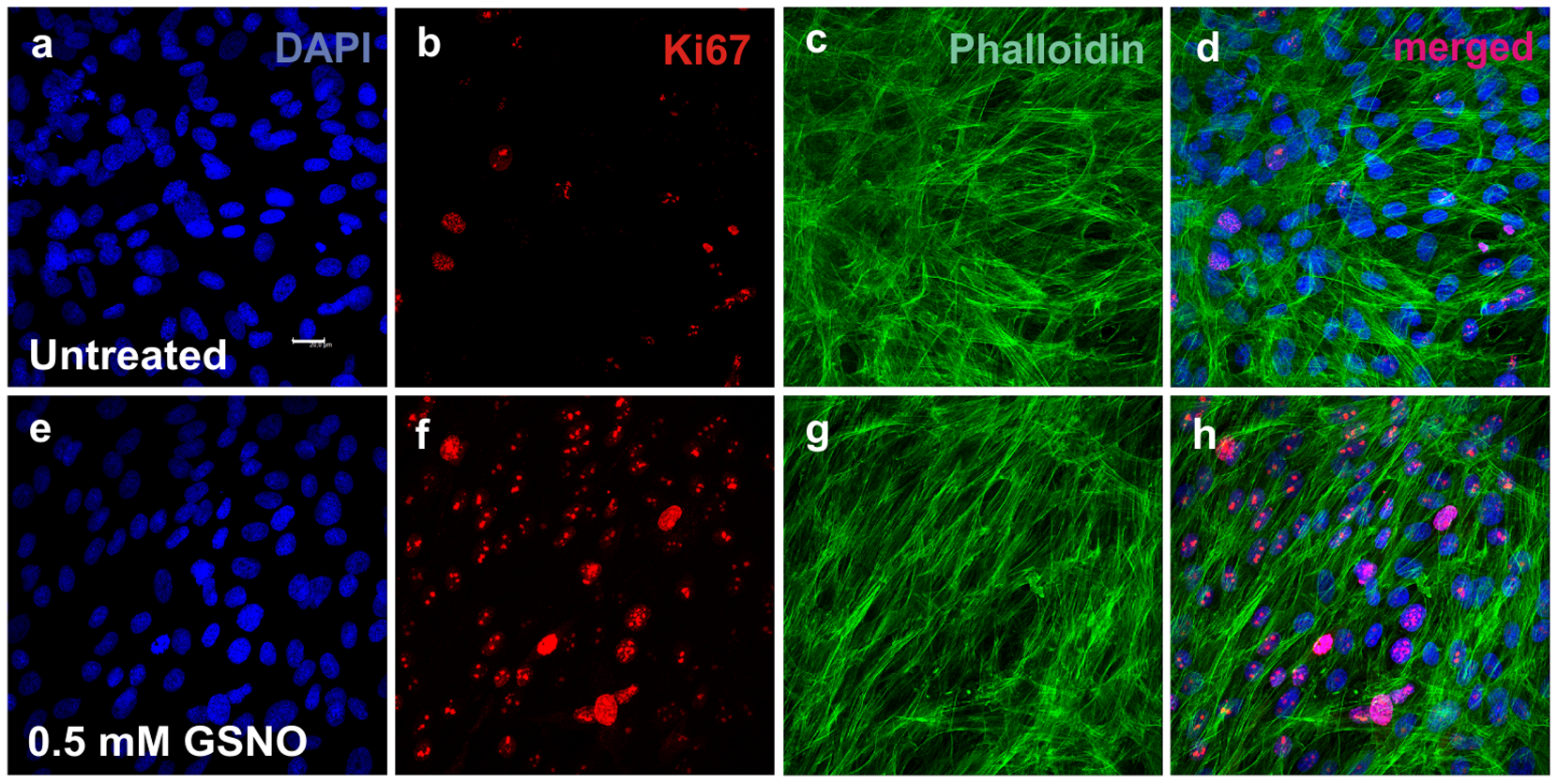
Primary cultures of oral fibroblasts from newborn rats incubated for 90 min in medium containing GSNO (a–d) or GSNO-free (e–h). The cells were immunostained using antibodies against smooth muscle anti-alpha-actin (c, d, g and h) or Ki67 (b, d, f and h), and nuclei were labeled with DAPI (a, d, e and h). Treatment of cells with 0.5 mM HPMC/GSNO for 90 min increased the expression of Ki67 (f and h) compared with untreated cells (b and d). Merged images are shown on the right (d and h). Scale bar = 10 µm.

**Figure 9:**
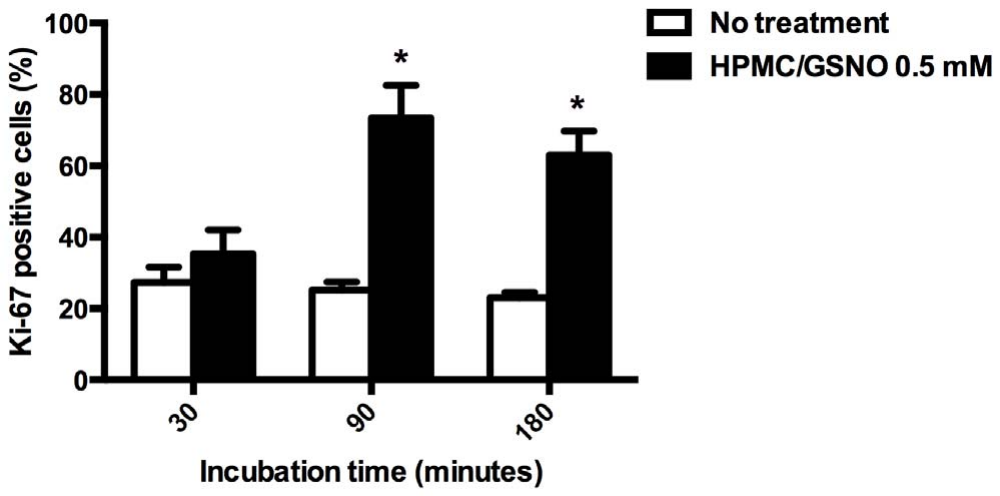
Quantification of immunohistochemical staining of oral myofibroblasts using an anti-Ki67 antibody. A robust increase in Ki67-positive cells was observed following treatment with S-nitrosoglutathione (GSNO; 0.5 mM) for 90 and 180 min. Values denote the means ± standard deviations. * denotes a significant difference (P<0.05) compared with untreated cells.

Figure 8 shows representative immunostaining for Ki67, a marker of proliferating cells, in myofibroblasts incubated for 90 min in medium containing GSNO (0.5 mM) (**Figures 8e, 8f, 8g, 8h**) or that was GSNO-free (**untreated control;** **Figures 8a, 8b, 8c and 8d**). An increase in the expression of Ki67 was observed in the cells incubated with GSNO (**0.5 mM;** **Figures 8b and 8d**) compared with untreated cells (**Figures 8f and 8h**). Figure 9 illustrates fibroblast proliferation (based on Ki67 expression) in the GSNO-treated cells and the untreated control cells after 30, 90 and 180 min of incubation. Treatment of cells with GSNO for 90 and 180 min, but not 30 min, resulted in an increase (P<0.05) in Ki67-positive cells compared with untreated control cells.

## Discussion

We demonstrated that the topical application of HPMC/GSNO formulations did not prevent lesions induced by 5-FU in the oral mucosa of hamsters on the 10^th^ day of experiment, which corresponds to maximal mucositis in hamsters [30]. However, our results showed that the topical application of 0.5 mM HPMC/GSNO accelerated the healing process, as observed on the 14^th^ day after initial administration of 5-FU. This positive effect of HPMC/GSNO on the healing phase of experimental oral mucositis was associated with reduced mucosa-inflammatory cell infiltration, decreased TNF-α levels and reduced immunostaining for iNOS and TNF-α in the cheek pouches of hamsters subjected to 5-FU-induced oral mucositis. In addition, in both *in vivo* and *in vitro* experiments, we also observed a positive effect of GSNO on cell proliferation based on Ki67 immunostaining. We did not observe anti-inflammatory or proliferative effects of 0.1 mM HPMC/GSNO on 5-FU-induced oral mucositis. We speculate that this concentration was not sufficient to provide effective concentrations of NO in the oral mucosal tissue. Additionally, under the same conditions, treatment with 2.0 mM HPMC/GSNO did not show any positive effects on the healing of oral ulcers. Consistent with our study, it has been demonstrated that high doses of exogenous NO increase clot formation and reactive tissue and impair collagen organization, which together contribute to cellular toxicity and delayed wound repair [31]. Our data are consistent with literature showing that the biological effect of NO is highly dependent on its concentration [32], [33].

In this respect, it must be noted that the sustained level of NO detected by chemiluminescence, shown in Figs. 1A and 1B, implies a constant rate of GSNO decomposition and NO release within the HPMC vehicle. This behavior may be explained by the bimolecular reaction between two GSNO molecules, based on Eq. 1:
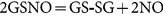
(1)where the only byproduct, in addition to NO, is oxidized glutathione (GS-SG). The straight lines in Figure 1B, suggesting a zero-order reaction, are in fact the result of the small change in GSNO concentration after 1 h of decomposition, allowing to assume that the GSNO concentration remains approximately constant during this time period. Thus, the rate of reaction 1 can be expressed as:
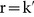
(2)where k′ = k[GSNO]^2^, k is the second-order rate constant of the reaction and [GSNO]^2^ is constant. The increase of only 5.7-fold in the rate of NO release (from 14 to 80 nmol/mL h) with the 4-fold increase in GSNO concentration (from 0.5 to 2.0 mM) reflects a restriction imposed by the viscous HPMC matrix on the diffusion of the GSNO molecules; thus, reducing the frequency of effective collisions that lead to NO production (based on reaction 1). This effect may underlie the slow release of NO from the HMPC/GSNO formulations that, in turn, is critical for obtaining therapeutic actions of NO during topical applications. It has been shown previously that the topical application of hydrogels containing GSNO was capable of promoting wound healing, leading to higher rates of wound contraction and re-epithelization, lower levels of inflammatory cells and an increase in collagen fiber density [18], [19], [34]. In these cases, the maximum GSNO concentration used in the hydrogels was 200 µM, which is 2.5-fold lower than the therapeutic GSNO concentration used in this current study. Together with these results, the current data showing an absence of therapeutic action of the 2.0 mM HPMC/GSNO formulation (with which the NO release rate was 5.7-fold higher), suggest that GSNO concentrations for topical therapeutic action in wound healing must remain in the micromolar range, most likely below 500 µM. Above this concentration, one may expect cytotoxic NO actions, although the threshold of cytotoxicity will necessarily depend on the type of tissue and the environment in which NO is delivered.

The increase in collagen content during wound repair observed in this current study may be attributed to an increase in collagen synthesis and/or proliferation of fibroblasts [35], [36]. Consistent with other experimental studies [34], [37], our results revealed an increase in HPMC/GSNO-mediated fibroblast proliferation, based on Ki67 immunostaining, and an increase in collagen deposition (data not shown) in the cheek pouches of hamsters subjected to oral mucositis.

It is well established that fibroblasts play a significant role in various stages of the healing process. Following injury, fibroblasts migrate into the wound, proliferate and produce matrix proteins (fibronectin, hyaluronic acid, collagen and proteoglycans) [38]. In addition, under the influence of growth factors [39] and mechanical stress [40], fibroblasts differentiate into myofibroblasts characterized by the presence of stress fibers that contain α-smooth muscle actin [41]. In contrast, it has been reported that NO stimulates inflammatory cells to secrete a greater level of growth factors including TGF-β [42], which is known to stimulate myofibroblast differentiation. Ligands of the TGF-β superfamily bind to a type II TGF-β receptor (TGF-β RII), a serine/threonine receptor kinase, and in turn phosphorylate a type I TGF-β receptor [43]. Although there is a high affinity of type II receptors for TGF-β, their interaction is controlled by the availability of the active form of TGF-β [44]. We observed a significant increase in TGF-β RII in the group of hamsters subjected to 5-FU-induced oral mucositis and treated with 0.5 mM HPMC/GSNO compared with untreated animals. Denton et al. demonstrated that TGF-β and its receptors are critical for the wound healing process. Those authors showed that a lack of TGF-β RII in a transgenic mouse resulted in impaired wound healing [45]. Thus, we speculate that the positive effect of 0.5 mM HPMC/GSNO on mucosal wound healing observed in the current study may be partially due to an upregulation of the TGF-β pathway followed by an increase in myofibroblast differentiation, because myofibroblasts robustly promotes wound closure [44]. Consistent with this hypothesis, our *in vitro* studies showed a significant increase in the expression of Ki67 in myofibroblasts incubated with GSNO (0.5 mM) compared with the untreated cells.

Moreover, we demonstrated that 0.5 mM HPMC/GSNO significantly reduced the 5-FU-induced increase in TNF-α levels and expression in cheek pouch tissues on day 14. In contrast, we did not observe any significant differences in IL-1β levels or expression in cheek pouch tissue between groups subjected to 5-FU-induced oral mucositis. It is well established that cytokines, in particular TNF-α and IL-1 β, play important roles in the pathophysiology of oral mucositis [4], [30]. The literature indicates that inflammatory cytokines stimulate iNOS-derived NO production and that NO mediates cytokine cytotoxicity and inflammatory events [46]. Consistent with these findings, we detected a significant increase in immunostaining for iNOS in the inflamed connective tissue of hamster cheek pouches on the 14^th^ day of the 5-FU-induced oral mucositis when compared with the Healthy group. The topical application of 0.5 and 2.0 mM HPMC/GSNO inhibited iNOS expression indicating that GSNO potentially protects against chemotherapy-induced iNOS activation in oral mucositis [37], [47]. Consistent with our data, it has been shown that GSNO, by reducing iNOS, reduces TNF-α expression in a rat model of focal cerebral ischemia [48] and in experimental periodontal disease in rats [49]. The negative modulation of iNOS by GSNO may underlie these findings. Indeed, it is well established that NO, whether released from exogenous donors or generated enzymatically, can inhibit the expression and activity of iNOS [50].

In this study, the topical administration of HPMC/GSNO formulations to hamsters subjected to 5-FU-induced oral mucositis provided evidence that apart from its effect on proliferation, 0.5 mM HPMC/GSNO significantly decreased TUNEL-positive cells in cheek pouch tissue when compared with the Saline group suggesting an inhibitory effect on 5-FU-induced cell death. Cell death is considered an important component of chemotherapy-induced mucosal injury [5]. Both chemo- and radiotherapy damage the mucosal lining and induce apoptosis [5], [51]. However, the mechanism by which GSNO decrease TUNEL-positive cells requires further investigation.

An important aspect of this current study is the identification of periodontopathogens in the context of oral mucositis. Mucositis ulcers are deep and are colonized quickly by oral bacteria [5]. In animal models, the number of mucosal bacteria increases by over 300-fold during the transition from intact to ulcerated epithelium [5] suggesting that ulcerated mucosa is a desirable colonization site. In addition, individuals with periodontitis that present a more complex microbiota show a higher risk of developing oral mucositis, which reinforces the relevance of studying the role of bacteria in the pathophysiology of oral mucositis [7]. Thus, the current investigation analyzed the mean frequency of detection and the levels of oral periodontopathogens in samples collected from cheek pouches of animals subjected to 5-FU-induced mucositis and from healthy hamsters.

In this study, increases in Gram-negative bacteria were observed in animals subjected to oral mucositis compared with healthy animals. Consistent with these results, Wang et al. reported that chemotherapy favors the growth of Gram-negative anaerobic bacteria with virulent phenotypes, leading to a cascade of inflammatory events and triggering oral mucositis [52]. We also observed that the topical application of 0.5 mM HPMC/GSNO greatly reduced the levels of several periodontopathogens including *P. gingivalis* and *T. forsythia*. This finding is very relevant given that *P. gingivalis* is considered one of the main pathogens in the oral cavity because it is resistant to nitrosative stress [53], [54]. Furthermore, it has been shown that *P. gingivalis* and associated secreted products inhibit the migration of oral epithelial cells *in vitro* [55] suggesting their involvement in delayed wound healing.

## Conclusions

In this investigation, we demonstrated that 0.5 mM HPMC/GSNO accelerated the healing of lesions induced by 5-FU in the oral mucosa of hamsters on day 14, most likely due to its anti-inflammatory, antimicrobial, anti-apoptotic and proliferative effects.

## Supporting Information

File S1 Figure S1, Real-time NO release profile of the 2.0 mM HPMC/GSNO formulation measured by chemiluminescence at 37°C. Inset: Integrated NO signal extracted from the kinetic curve, showing the total NO released from the formulation over the same time-scale. The straight lines show the linear regression of the experimental data and the calculated rates of NO release for two sections of the curve. Figure S2, The cheek pouches of animals subjected to 5-FU-induced oral mucositis showed a significant increase (P<0.05) of neutrophils on day 14 when compared with the Healthy and Mechanical Trauma (MT) control groups. 0.5 mM HPMC/GSNO treatment substantially (P<0.05) reduced the number of neutrophils in cheek pouch tissue compared with both the HPMC and Saline groups. Bars denote the means ± standard errors of the number of neutrophils from at six slides per group (6 animals per group). *denotes a significant difference (P<0.05) compared with the Healthy group; **denotes a significant difference (P<0.05) compared with the MT group, +denotes a significant difference (P<0.05) compared with the Saline group; #denotes a significant difference (P<0.05) compared with the HPMC group. Data were analyzed using the Kruskal Wallis and Mann Whitney tests.
(DOC)
