# Supplementary material for: S-Nitrosoglutathione Accelerates Recovery from 5-Fluorouracil-Induced Oral Mucositis
Source: PLoS One. 2014 Dec 5;9(12):e113378. doi: 10.1371/journal.pone.0113378 (PMC4257535; doi:10.1371/journal.pone.0113378)
Supplement: File S1 — Figure S1, Real-time NO release profile of the 2.0 mM HPMC/GSNO formulation measured by chemiluminescence at 37°C. Inset: Integrated NO signal extracted from the kinetic curve, showing the total NO released from the formulation over the same time-scale. The straight lines show the linear regression of the experimental data and the calculated rates of NO release for two sections of the curve. Figure S2, The cheek pouches of animals subjected to 5-FU-induced oral mucositis showed a significant increase (P<0.05) of neutrophils on day 14 when compared with the Healthy and Mechanical Trauma (MT) control groups. 0.5 mM HPMC/GSNO treatment substantially (P<0.05) reduced the number of neutrophils in cheek pouch tissue compared with both the HPMC and Saline groups. Bars denote the means ± standard errors of the number of neutrophils from at six slides per group (6 animals per group). *denotes a significant difference (P<0.05) compared with the Healthy group; **denotes a significant difference (P<0.05) compared with the MT group, +denotes a significant difference (P<0.05) compared with the Saline group; #denotes a significant difference (P<0.05) compared with the HPMC group. Data were analyzed using the Kruskal Wallis and Mann Whitney tests. (DOC) [file pone.0113378.s001.doc]

Supporting material to the article:

**S-nitrosoglutathione accelerates recovery from 5-fluorouracil-induced oral mucositis**

Skeff MA^1,2^, Brito GA^2^, de Oliveira MG^5^, Braga CM^2^, Cavalcante MM^2^, Baldim V^5^, Afonso RCH^1^, Silva-Boghossian CM^3^, Colombo AP^3^, Ribeiro RA^4^, Moura-Neto V^1^*, Leitão RF^2^*

*^1^Laboratory of Cell Morphogenesis, Institute of Biomedical Sciences, Federal University of Rio de Janeiro,*

*^2^* *Department of Morphology, School of Medicine, Federal University of Ceará,*

*^3^* *Institute of Microbiology, Federal University of Rio de Janeiro,*

*^4^* *Department of Physiology and Pharmacology, School of Medicine, Federal University of Ceará,*

^5^ *Institute of Chemistry, University of Campinas, UNICAMP, Campinas, SP, Brazil.*

^*^Co-corresponding authors: Moura-Neto V (vivaldo@icb.ufrj.br), Leitão RF (leitao_renata@yahoo.com.br)


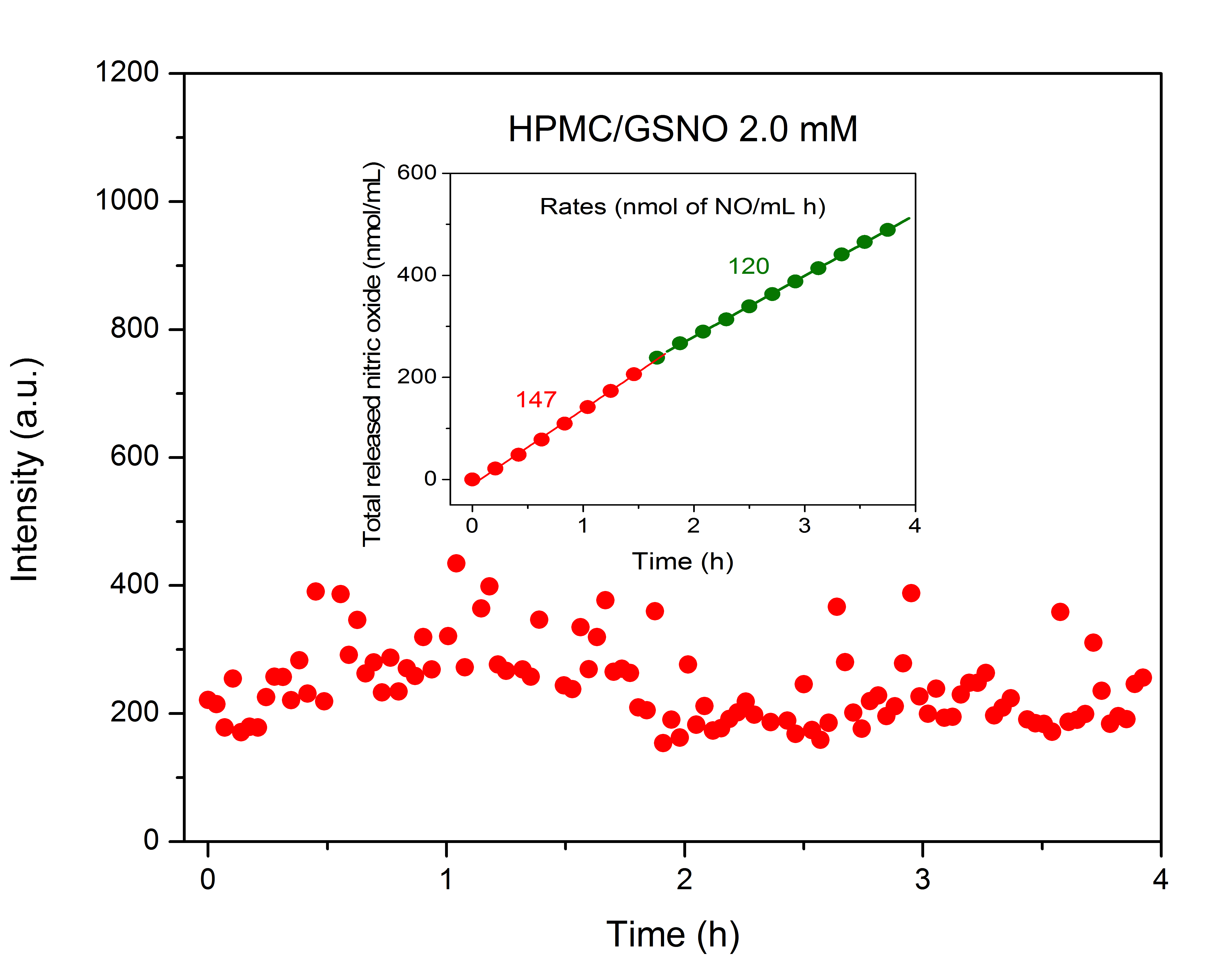


**Figure S1**. Real-time NO release profile of the 2.0 mM HPMC/GSNO formulation measured by chemiluminescence at 37 °C. Inset: Integrated NO signal extracted from the kinetic curve, showing the total NO released from the formulation over the same time-scale. The straight lines show the linear regression of the experimental data and the calculated rates of NO release for two sections of the curve.

**Experimental details**:

The profile of NO release from the HPMC/GSNO formulations 2.0 mM was determined over a period of more than 4 h using a chemiluminescence NO analyzer (NOA 280i, Sievers Instruments Inc., Boulder, CO, USA) operated at 37 °C with an O_2_ pressure of 6.0 psig and a N_2_ pressure of 6.0 Torr. A volume of 2.0 mL of the HPMC/GSNO formulation, containing 100 µL of an anti-foaming solution (provided by Sievers) was used in the measurement.


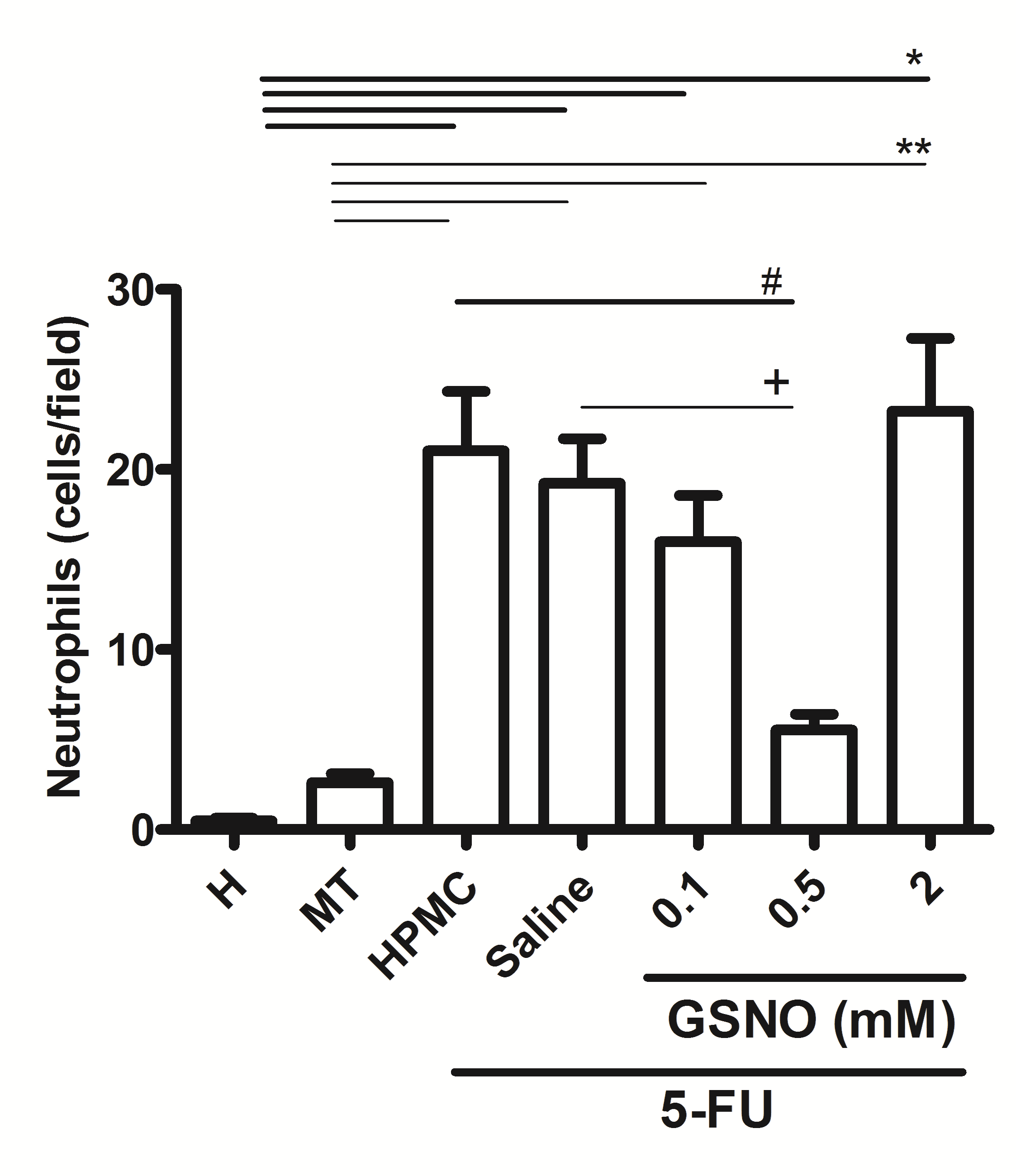


**Figure S2:** The cheek pouches of animals subjected to 5-FU-induced oral mucositis showed a significant increase (P<0.05) of neutrophils on day 14 when compared with the Healthy and Mechanical Trauma (MT) control groups. HPMC/GSNO 0.5 mM treatment substantially (P<0.05) reduced the number of neutrophils in cheek pouch tissue compared with both the HPMC and Saline groups. Bars denote the means ± standard errors of the number of neutrophils from at six slides per group (6 animals per group). *denotes a significant difference (P<0.05) compared with the Healthy group; **denotes a significant difference (P<0.05) compared with the MT group, +denotes a significant difference (P<0.05) compared with the Saline group; #denotes a significant difference (P<0.05) compared with the HPMC group. Data were analyzed using the Kruskal Wallis and Mann Whitney tests.

**Experimental details**: Oral mucositis was induced in hamsters by intraperitoneal (i.p.) injection of 5-FU followed by mechanical trauma (MT) of the cheek pouch. Animals received topical applications of a gel containing S-nitrosoglutathione (HPMC/GSNO 0.1, 0.5 and 2 mM) 30 min prior to 5-FU and twice daily for 14 days. Control groups comprised normal animals (N), animals subjected to mechanical trauma (MT) only and animals subjected to 5-FU-induced oral mucositis that received local application of saline (saline) or vehicle (HPMC). The animals were euthanized on the 14^th^ day after the initial injection of 5-FU and cheek pouch samples were harvested, fixed in 10% neutral buffered formalin, dehydrated and embedded in paraffin. Sections of 5 µm thickness were obtained for hematoxylin-eosin staining (H&E). The neutrophils were counted (10 fields per slide/6 animals per group; X1000) in order to perform a statistical comparison.
